# Supplementary material for: Structure of full-length ERGIC-53 in complex with MCFD2 for cargo transport
Source: Nat Commun. 2024 Mar 16;15:2404. doi: 10.1038/s41467-024-46747-1 (PMC10944485; doi:10.1038/s41467-024-46747-1)
Supplement: Supplementary file 1 — Supplementary Information [file 41467_2024_46747_MOESM1_ESM.pdf]

Structure of full-length ERGIC-53 in complex with MCFD2 for cargo transport

Satoshi Watanabe[1,2,3*], Yoshiaki Kise[4], Kento Yonezawa[5,8], Mariko Inoue[1], Nobutaka Shimizu[5], Osamu Nureki[4] and Kenji Inaba[1, 2,3,6,7*]

1: Institute of Multidisciplinary Research for Advanced Materials, Tohoku University, Sendai, Miyagi 980-8577, Japan.
2: Department of Molecular and Chemical Life Sciences, Graduate School of Life Sciences, Tohoku University, Sendai, Miyagi 980-8577, Japan.
3: Department of Chemistry, Graduate School of Science, Tohoku University, Sendai, Miyagi 980-8578, Japan.
4: Department of Biological Sciences, Graduate School of Science, The University of Tokyo, Bunkyo-ku, Tokyo, 113-0033, Japan
5: Structural Biology Research Center, Institute of Materials Structure Science, High Energy Accelerator Research Organization (KEK), Tsukuba, Ibaraki 305-0801, Japan
6: Medical Institute of Bioregulation, Kyushu University, Fukuoka 812-8582, Japan.
7: Core Research for Evolutional Science and Technology (CREST), Japan Agency for Medical Research and Development (AMED), Tokyo, Japan
8: Present address: Center for Digital Green-innovation, Nara Institute of Science and Technology, Ikoma, Nara 630-0192, Japan

*Correspondence
Satoshi Watanabe: satoshi.watanabe.c1@tohoku.ac.jp
Kenji Inaba: kenji.inaba.a1@tohoku.ac.jp

Supplementary information

15 supplementary figures
3 Supplementary tables

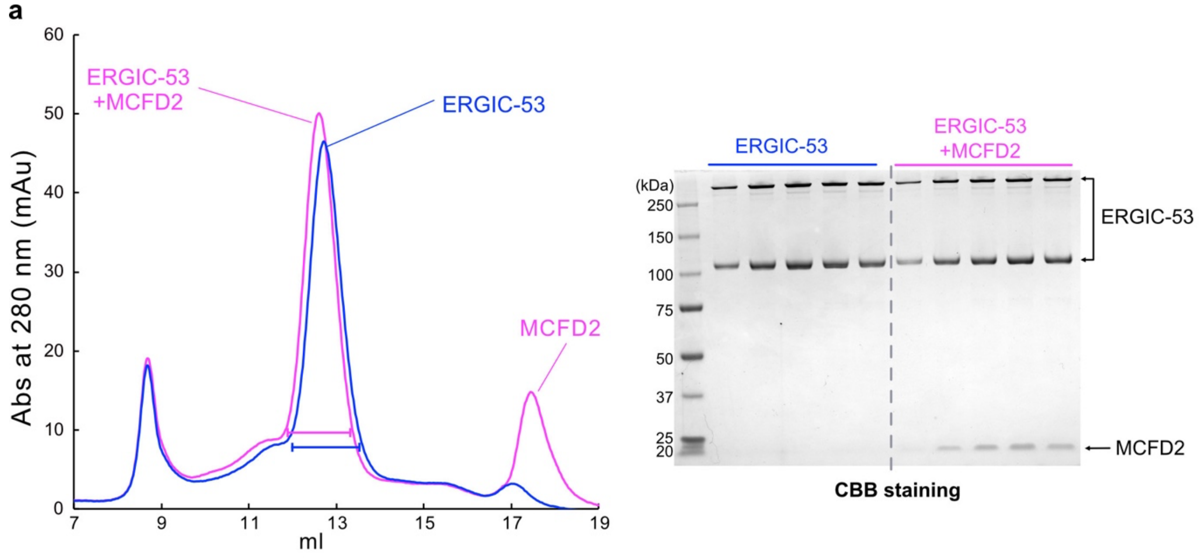

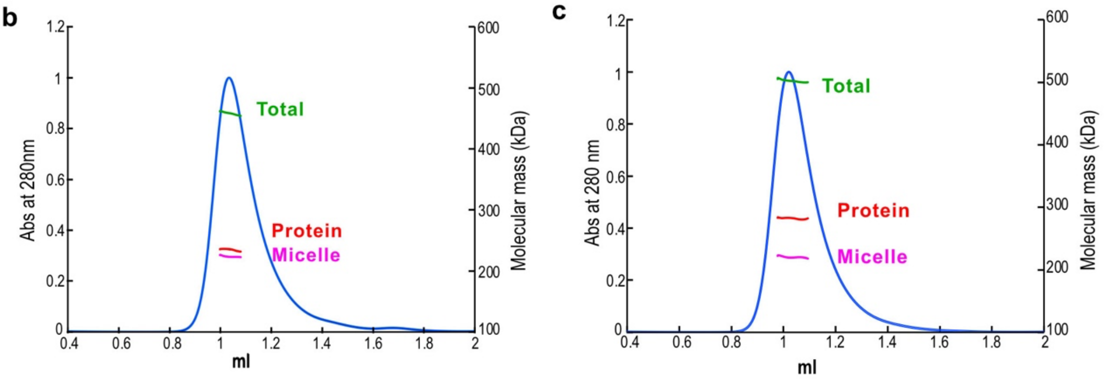

d

Molecular masses (kDa) determined by SEC-MALS

	ERGIC-53	ERGIC-53 +MCFD2
Protein	235	282
(tetramer)	(221.2)	(277.2)
(hexamer)	(331.8)	(415.8)
Micelle	223	220
Total	458	502

h

	ERGIC-53	ERGIC-53 +MCFD2
R_g (from Guinier)	101.5 Å	102.8 Å
R_g (from Pr)	108.7Å	110.8Å
D_{max}	347Å	350Å

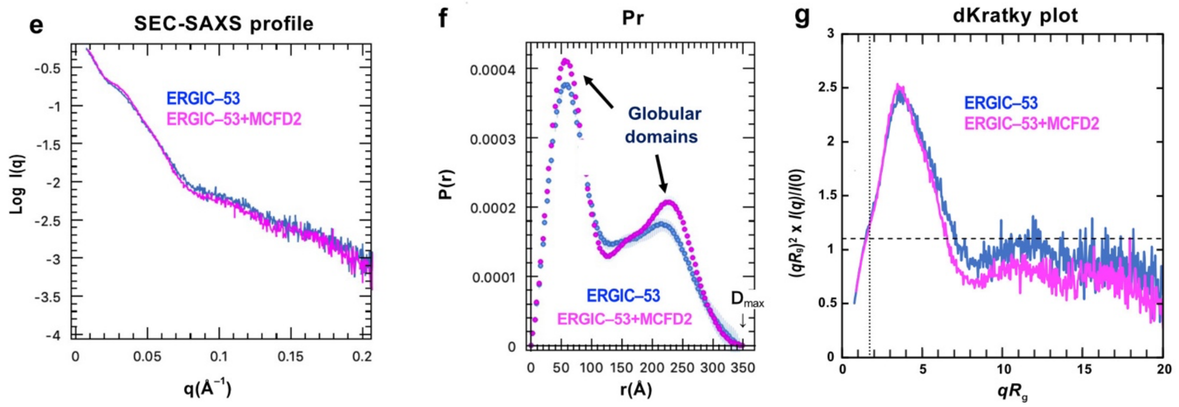

Supplementary Fig. 1 SEC-MALS/SAXS analysis of recombinant human ERGIC-53 in complex with MCFD

a) SEC profiles of purified ERGIC-53 (blue) and the mixture of ERGIC-53 and MCFD2 (pink). The right panel shows non-reducing SDS-PAGE analysis of each peak fraction, detected by CBB staining.

b,c) The SEC-MALS profile of ERGIC-53 (a) and its complex with MCFD2(b). Masses of the peak fractions determined by SEC-MALS analysis are indicated by the green line for total mass, red for protein mass and magenta line for micelle, respectively.

d) Summary of the determined molecular masses.

e) Experimental SAXS profiles of ERGIC-53 (blue) and ERGIC-53 in complex with MCFD2 (magenta).

f) Pair distance distribution functions P(r) of ERGIC-53 (blue) and the complex (magenta) determined from the SAXS profiles.

g) The dimensionless Kratky plots of ERGIC-53 (blue) and the complex (magenta) determined from the SAXS profiles. The dotted and dashed lines represent the positions of √3 and 3/e, respectively, and the peak appears at this cross point for a typical globular protein.

h) Summary of radius of gyration (R_g) and maximum dimension D_{max}.

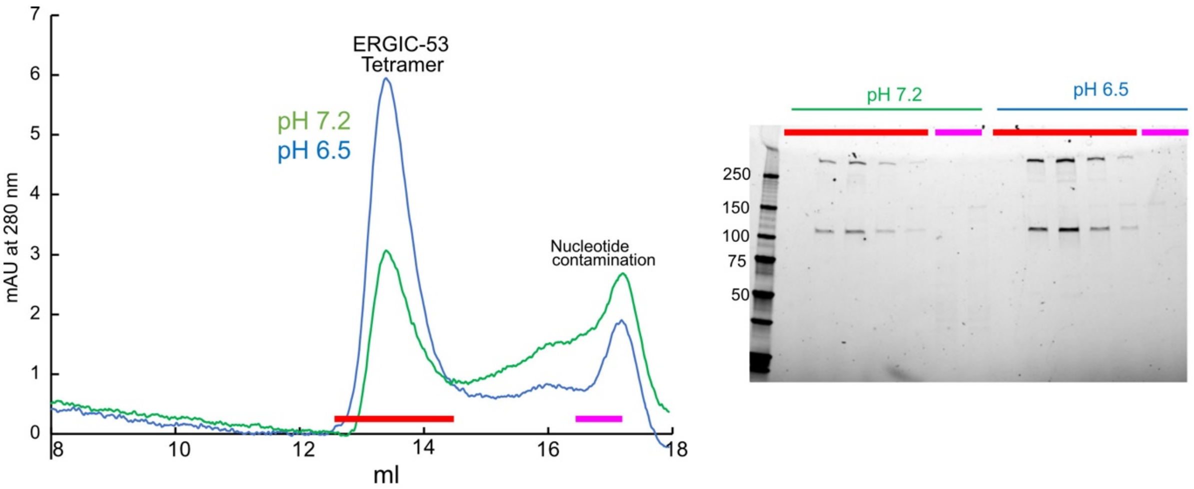

Supplementary Fig. 2 SEC- analysis of recombinant human ERGIC-53 at pH 7.2 and 6.2

SEC profiles of ERGIC-53 purified at pH 7.2 (green) and pH 6.5 (blue). The right panel shows SDS-PAGE analysis of peak fractions indicated by red and magenta, detected by stain-free technology. Note: The broad peaks observed at the larger elution volume (16-18 ml) were likely ascribed to the nucleotide contamination, because no protein bands were observed for these elution fractions in the SDS-PAGE analysis.

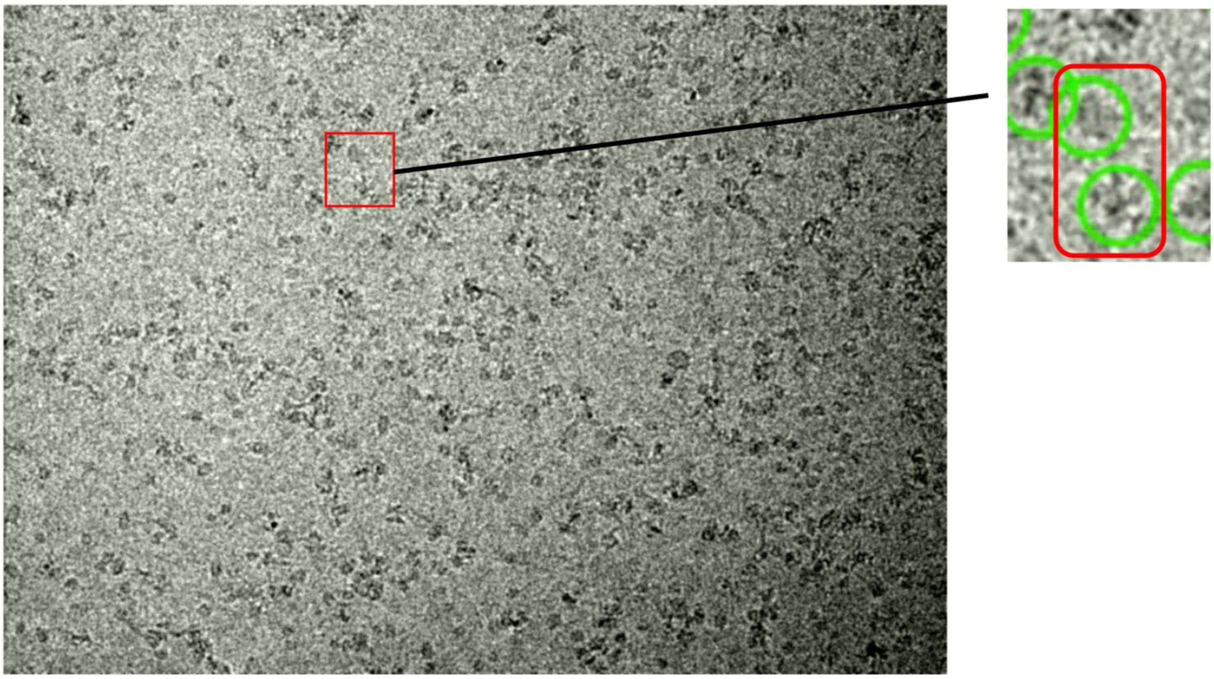

Supplementary Fig. 3 Cryo-EM analysis of ERGIC-53 in complex with MCFD2

A representative motion-corrected micrograph of ERGIC-53 with MCFD2. The inset shows a close-up view of a dumbbell-like particle of full-length ERGIC-53, indicated by a red square. Green circles represent particles picked by blob-based autopicking.

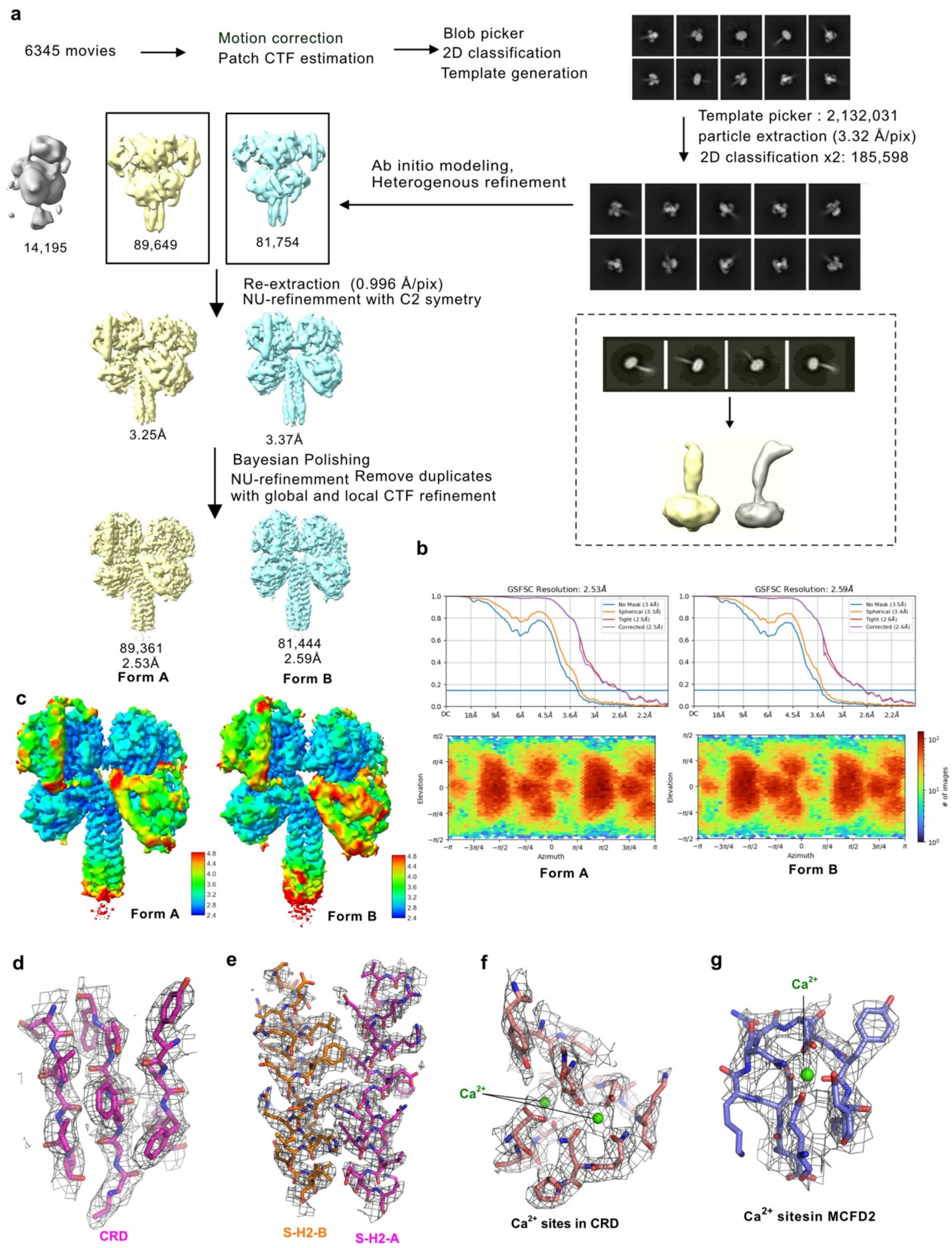

Supplementary Fig. 4 Cryo-EM image processing of the head region of ERGIC-53 in complex with MCFD2

a) Workflow of the image processing of each globular region. Representative 2D class average images and refined maps at each step are shown.

b) Gold standard Fourier shell correlation (GSFSC) resolution plots (upper panels) and Euler angle distributions (lower panels) of the final maps of form A and B, calculated with cryoSPARC.

c) Local resolution of the final maps calculated with the local resolution tool in cryoSPARC.

d~g) Representative EM density maps and models around (d) the center of CRD, (e) the central four-helix coiled coil, (f) Ca2+ binding site in the CRD, and (g) Ca binding site in MCFD2.

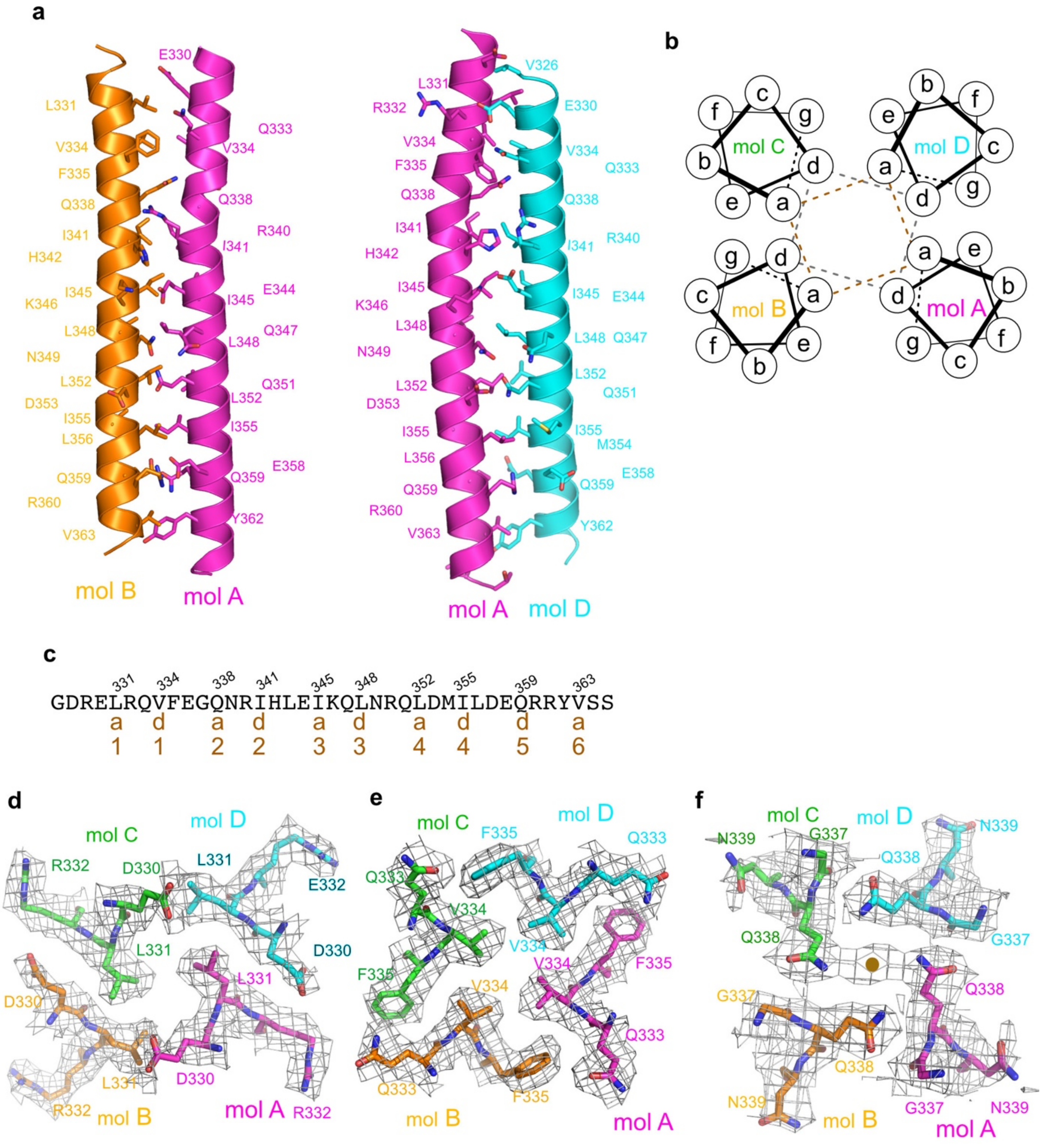

Supplementary Fig. 5 Details of the interfaces of the four-helix coiled coil formed by Stalk helix 2 (S-H2)

a) Details of the interactions between stalk helices 2 of ERGIC-53. b) Helical wheel projection of the four-helix coiled coil of S-H2. Heptad positions are labeled **a** to **g**. c) Amino acid sequence of S-H2. Residues at '**a**' and '**d**' heptad positions analyzed by SamCC are labeled. d-f) Cross sections of the coiled coil in d) L331 layer ('**a1**' layer); e) V334 layer ('**d1**' layer); and f) Q338 layer ('**a2**' layer) with EM maps (gray meshes). A brown sphere represents a bound water molecule or some ion.

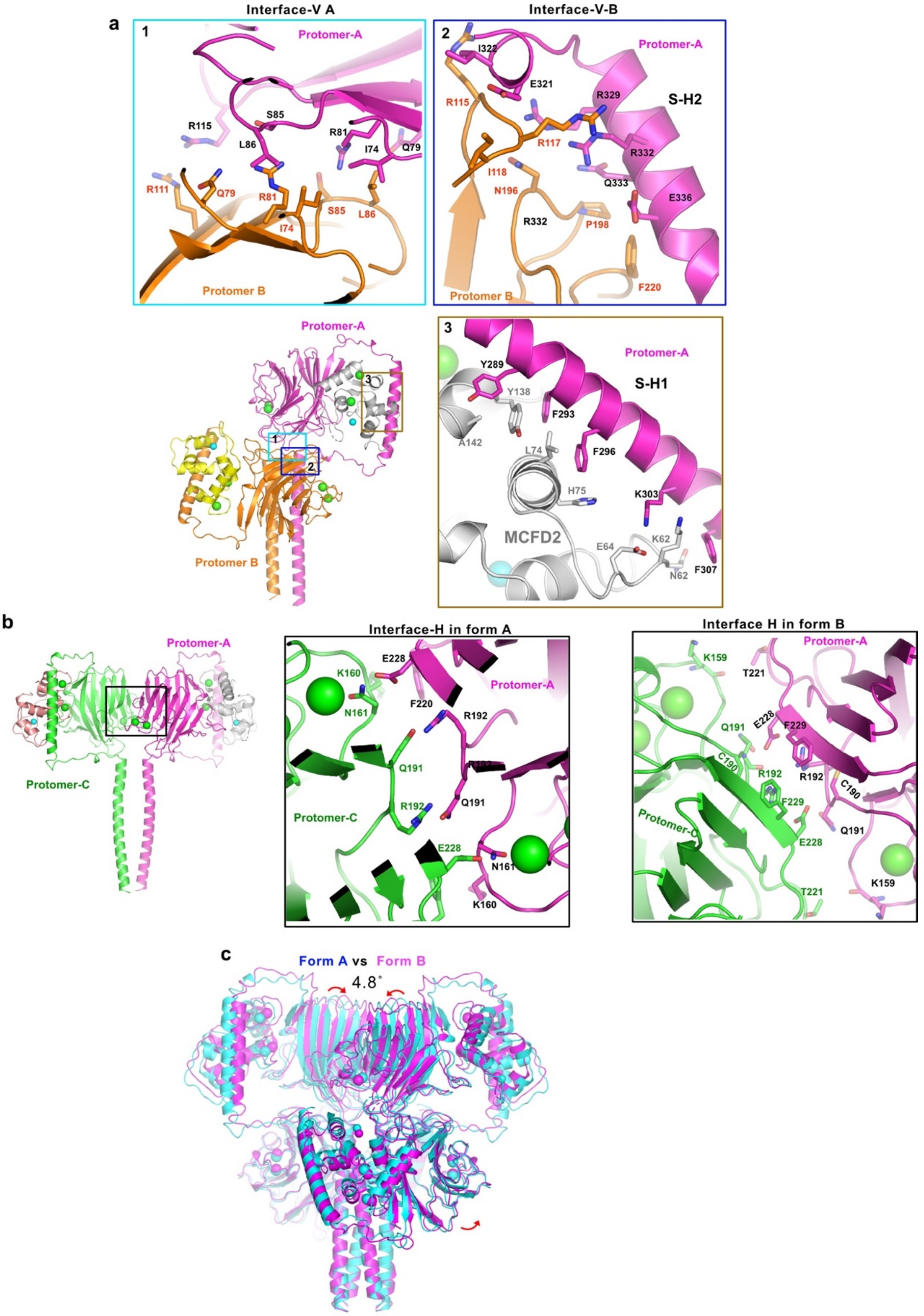

Supplementary Fig. 6 Details of domain interfaces

a) Details of the vertical interface (interface-V) and SH-1-MCFD2 interaction. The left lower panel shows the overall structure of the head region dimer. The other three panels show close-up views at the cyan, blue,

and yellow boxes, respectively; 1: interface between the upper and lower CRD; 2: interface between the lower CRD and S-H2 helix; 3: interaction between S-H1 and MCFD2. Residues involved in the interface are shown in stick models.

b) Details of the horizontal interface (interface-H). (left) The head region dimer formed by interface-H between the upper CRDs in form A; (middle and right) close-up view a of the interface H at the black box in form A (left) or form B (right). Residues involved in the interface are shown in stick models. Bound calcium ions are represented by green spheres.

c) Comparison of the form A (cyan) and form B (magenta) structures, in which their the central-coiled coils are superimposed on each other.

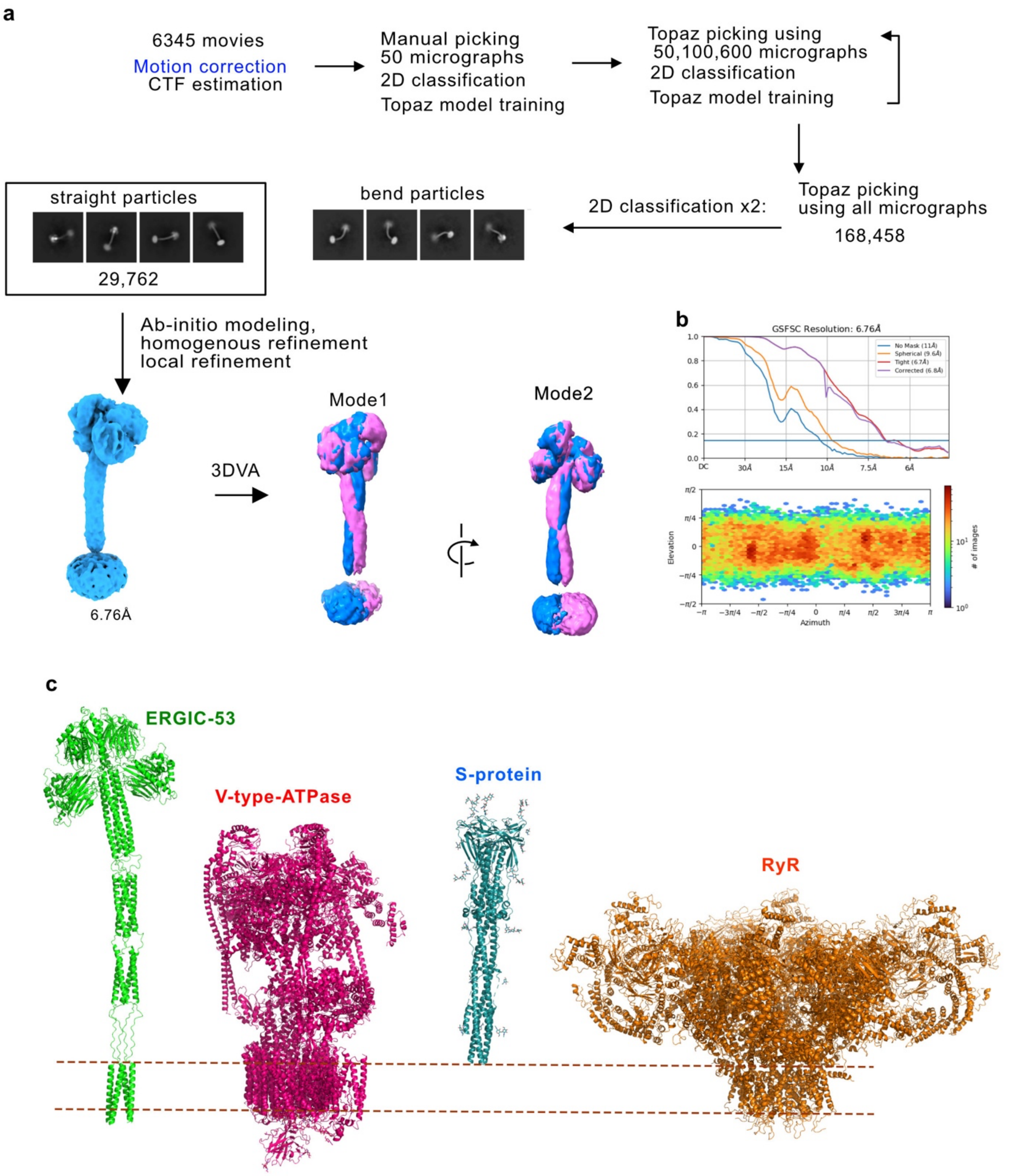

Supplementary Fig. 7 Imaging processing of the full-length particles of ERGIC-53.

a) Workflow of the image processing of the full-length ERGIC-53 particles. Representative 2D class average images and refined maps at each step are shown.

b) GSFSC resolution plots (upper panels) and Euler angle distributions (lower panels) of the final map at 6.7Å resolution.

c) Comparison of the molecular height of full length ERGIC-53 complexed with MCFD2 (this study) with those of V-type ATPase (PDB: 6WM2), S-protein from SARS-COV-2 (PDB: 6XRA), and RyR (PDB: 5TB4).

a Domain picking of ERGIC-53 without MCFD2

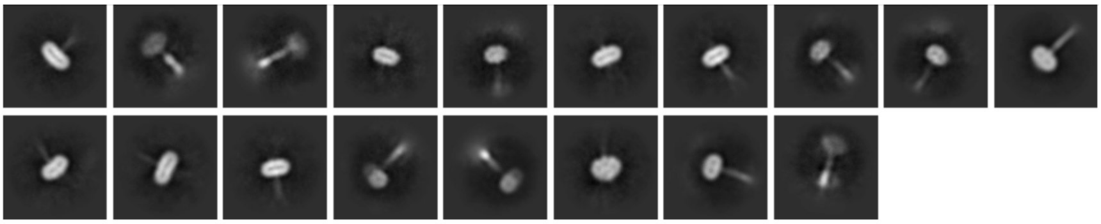

b Topaz picking of ERGIC-53 without MCFD2

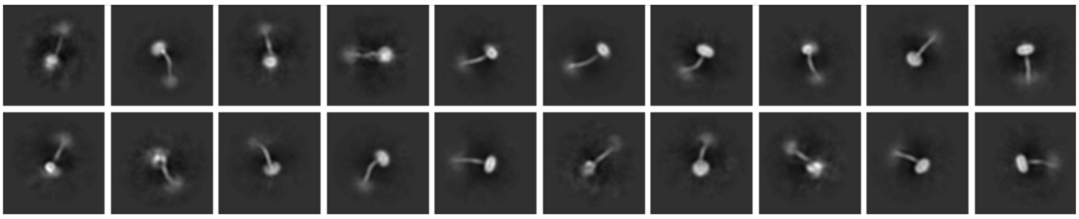

Supplementary Fig. 8 Cryo-EM SPA analysis of ERGIC-53 without MCFD2

Representative 2D class average images of the half regions of the particle(upper) and the full-length particles (lower).

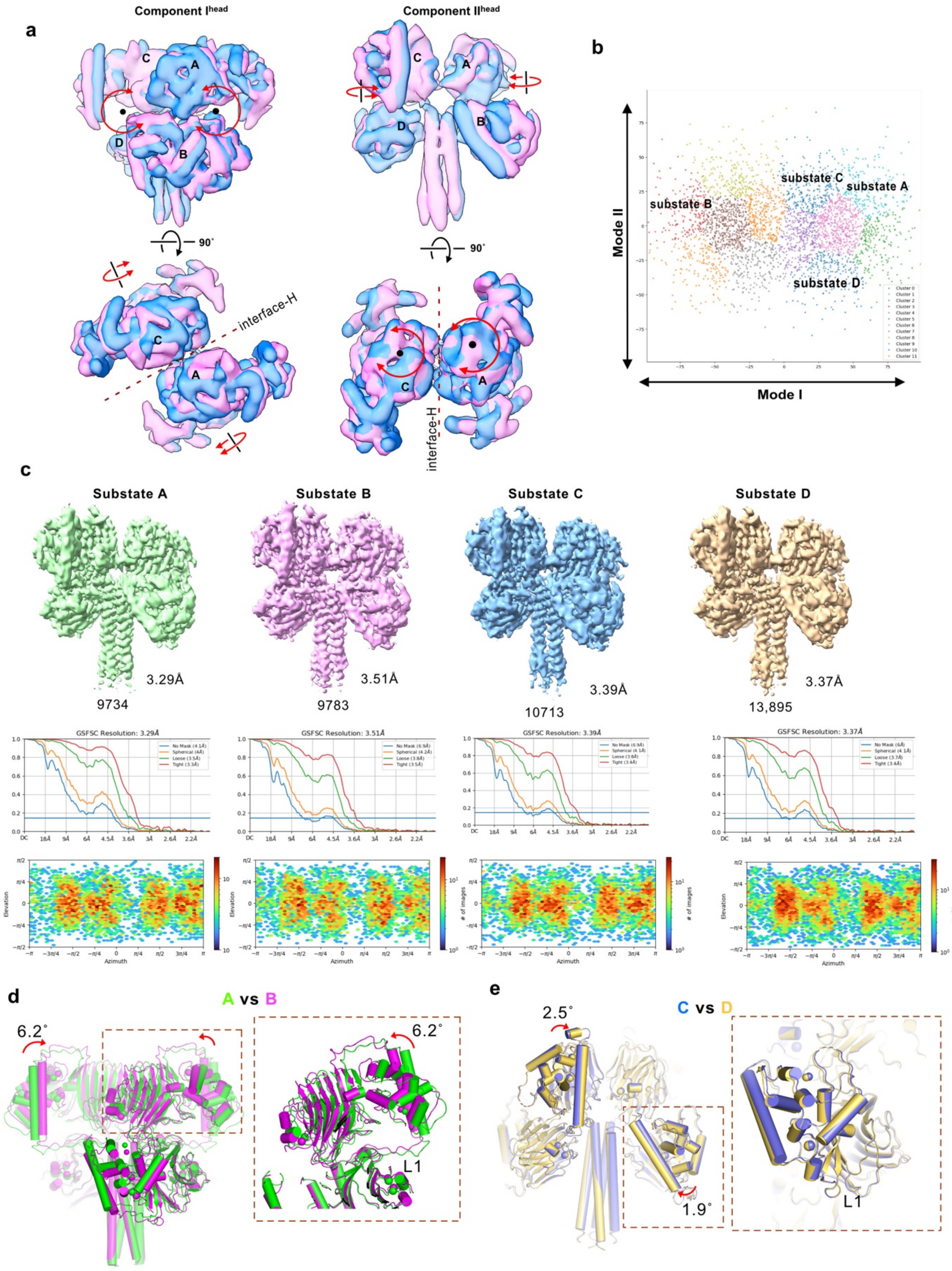

Supplementary Fig. 9 3DVA of the head region

a) Results of 3DVA of the head region with two variability components I and II. The EM maps of the first (pink) and last frames (blue) of continuous conformational changes generated by 3DVA are displayed. Arrows represent the rigid body rotation of each unit.

b) Each dot represents a particle plotted according to the 3DVA clustering.

c) Reconstruction and refinement of four substate structures of the head region. Particles belonging to each group (A, B, C, D) are indicated in the cluster plots in a. GSFSC resolution plots and Euler angle distributions of each substate structure are provided in the middle and lower panels, respectively.
d) Comparison between substates A and B.
e) Comparison between substates C and D.

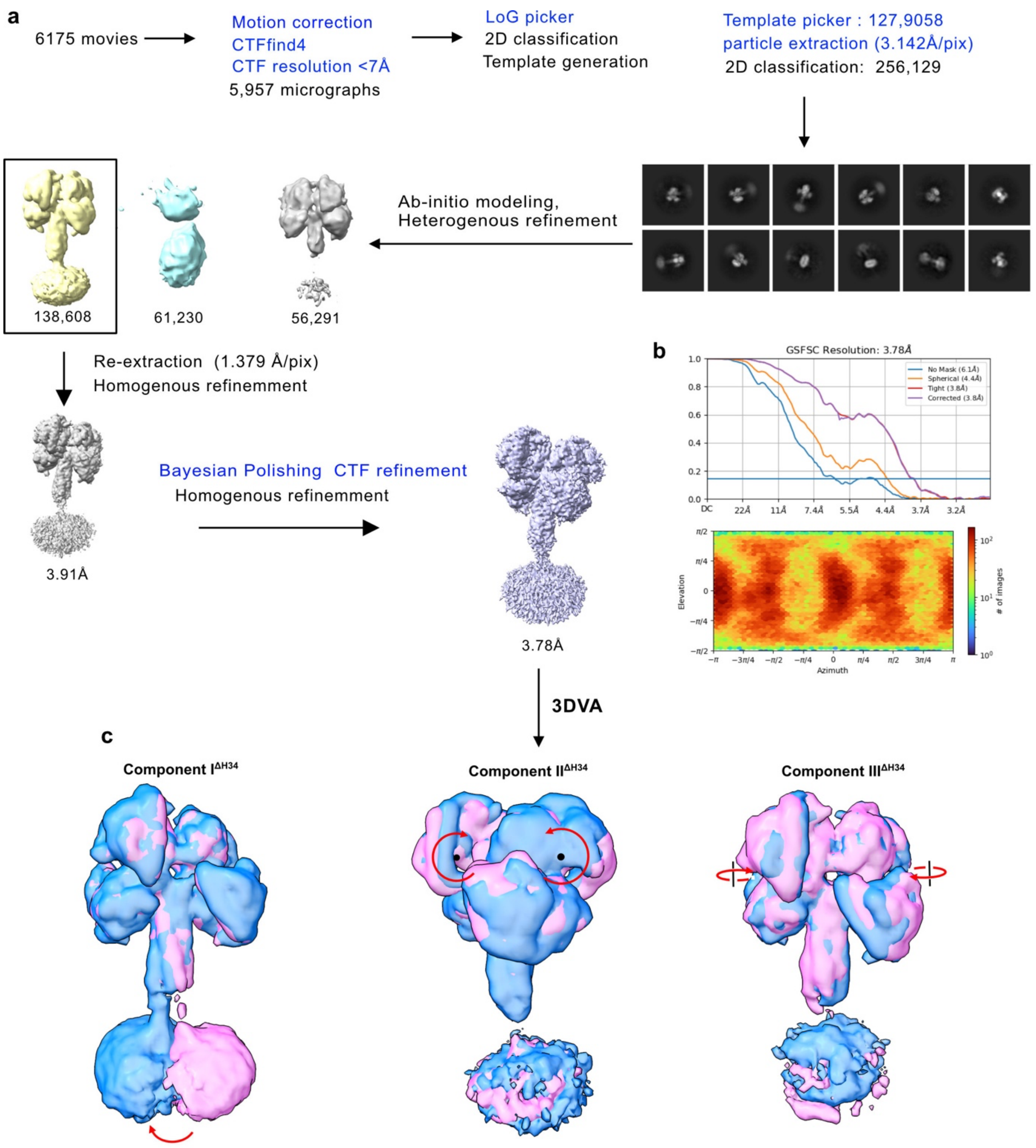

Supplementary Fig. 10 Cryo-EM image processing of the ERGIC-53 ΔH34 mutant with MCFD2

a) Workflow of the image processing of the ΔH34 mutant or ERGIC-53. Representative refined maps at each step are shown.

b) GSFSC resolution plots (upper panels) and Euler angle distributions (lower panels) of the final map at 3.78 Å resolution.

c) The results of 3DVA of this mutant with three variable components I, II and III. The EM maps of the first (pink) and last frames (blue) of continuous conformational changes generated by 3DVA are displayed. Arrows represent rigid body rotation of each unit.

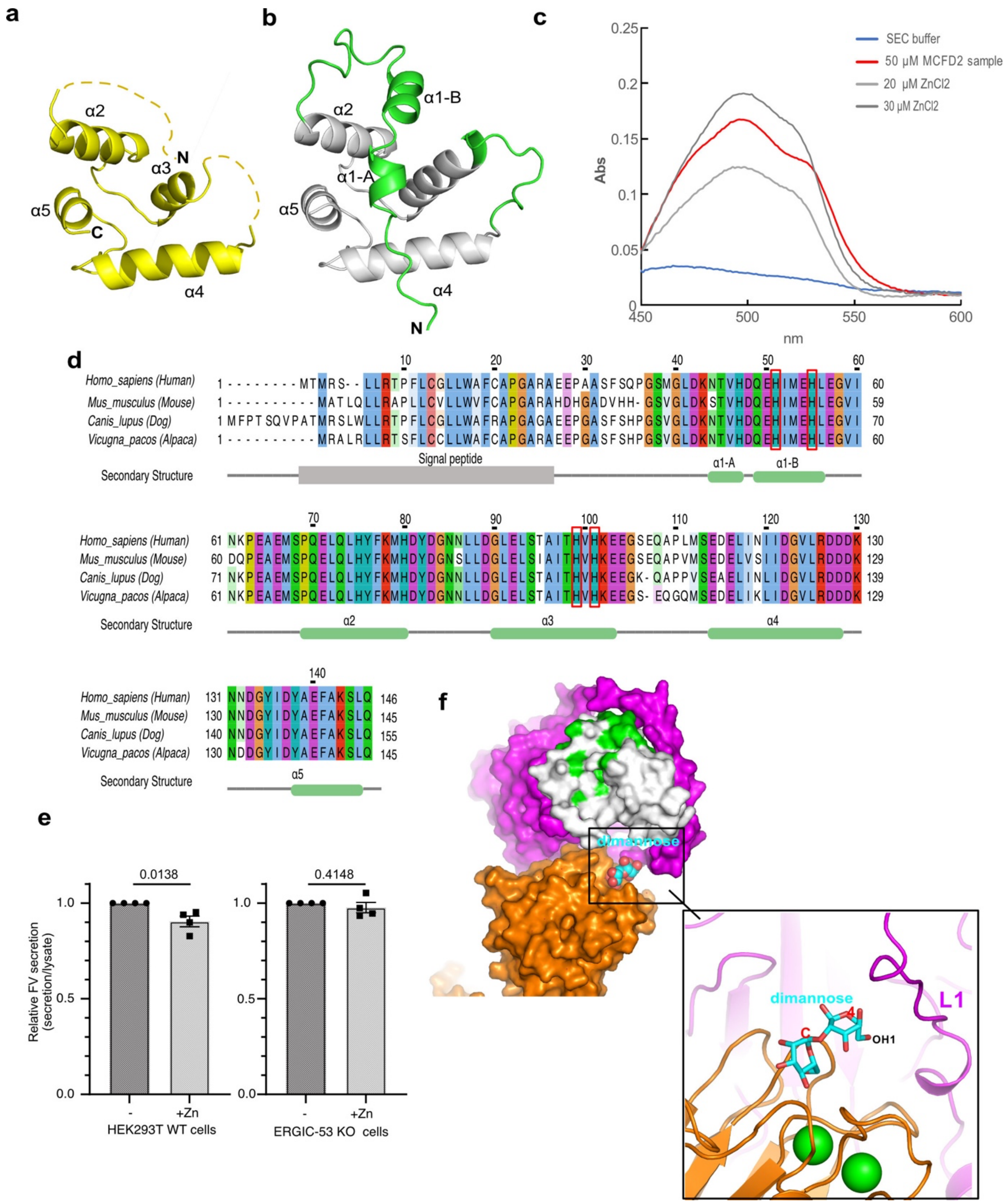

Supplementary Fig. 11 Details of Zn^{2+}-dependent regulation of MCFD2

a) The original crystal structure of MCFD2 (PDB: 4YGE)

b) A predicted structure of MCFD by Alphafold2. The residues that are missing in the original structures are shown in green.

c) UV-vis spectrum of the PAR assay of purified denatured MCFD2 (red) or buffer (blue).

d) Sequence alignment of MCFD2 homologues by Jalview. The four conserved Histidine residues are highlighted by red boxes.

e) Secretion assay of HiBiT-tagged FV in HEK293T cells or ERGIC-53 KO with or without zinc

supplementation. The amounts of secreted FV relative to that of intracellular FV under Zn supplementation conditions were quantified with a commercial HiBiT-tagged protein detection reagent, and normalized to the control conditions. Dots Each symbol represents the individual data points. Error represents the standard deviation of the mean (N=4 biological replicates). An unpaired two-tailed t-test was used for statistical analysis.

f) A docking model of dimannose in the present structure. The bound di-mannose is docked based on superposition of the crystal structure of the CRD with di-mannose (PDB: 4GKX). An inset shows a close-up view of di-mannose binding on the CRD, suggesting that the putative Man(4) moiety of Man9(GlcNAc)2 glycan lies near the L1 loop between the S-H1 and S-H2 helices.

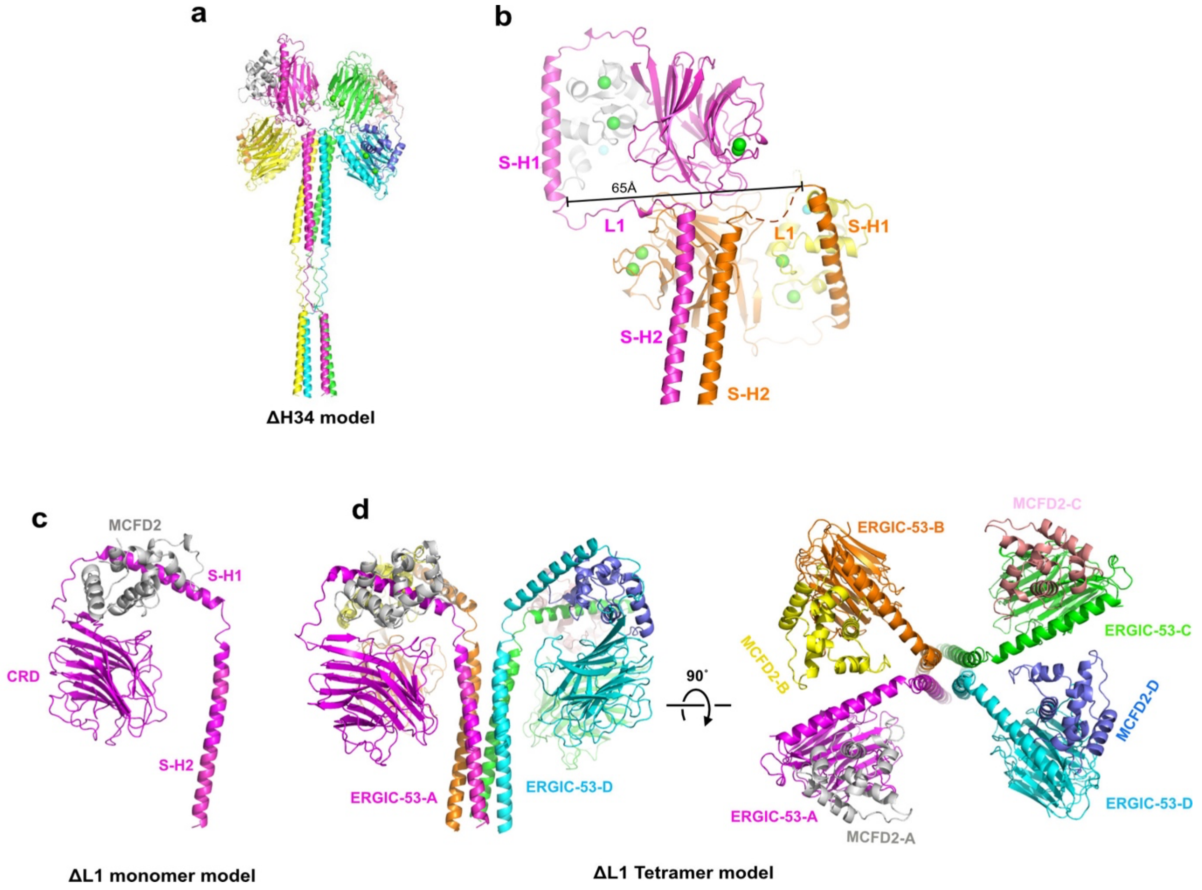

Supplementary Fig.12 Structure models of truncated mutant of ERGIC-53

a) Overall structure model of the ERGIC-53 ΔH34 mutant.

b) Close-up view of the L1 loop between two stalk helices (S-H1 and S-H2). In the present tetramer, the distance between the C-terminal ends S-H1 is ~65Å, apart by the L1 loop.

c) A model of the head region (CRD, SH1 and SH2) of the ΔL1 mutant monomer complexed with MCFD2 predicted by Colabfold v.1.5 with AF2

d) A tetramer model of the head region of the ΔL1 mutant with MCFD2 predicted by Colabfold v.1.5 with AF2 multimer

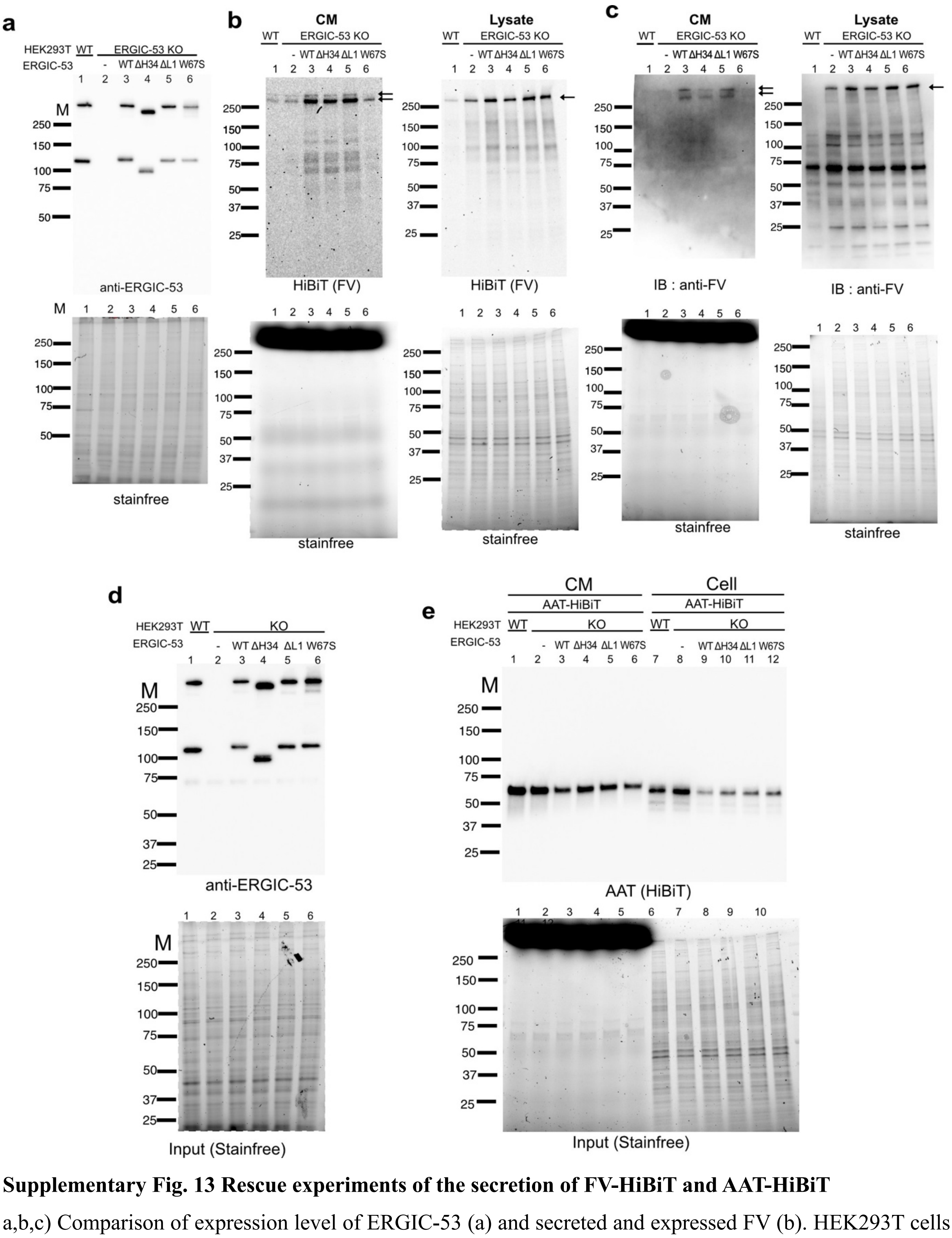

Supplementary Fig. 13 Rescue experiments of the secretion of FV-HiBiT and AAT-HiBiT

a,b,c) Comparison of expression level of ERGIC-53 (a) and secreted and expressed FV (b). HEK293T cells (Abcam) transfected with FV-HiBiT and empty vector, and ERGIC-53 KO 293T cells transfected with FV-HiBiT and ERGIC-53, indicated mutant or empty vector were incubated in the serum-free Expi293 medium for 4 h. Conditioned media and cell lysates were resolved by nonreducing SDS–PAGE (a) or reducing SDS–PAGE (b, c) and analyzed with immunoblotting with anti-ERGIC-53 antibody (a), LiBiT proteins for HiBiT tag (b) or anti-FV antibody (c). Total protein in each lane was detected by stain-free technology. Note: The transfection efficiency of FV in HEK293T WT cells (Abcam) were significantly lower than that in the KO

cells. Although the used anti-FV antibody failed to detect low levels of FV from these cells, HiBiT-blotting system showed higher sensitivity and detected secreted/expressed FV-HiBiT proteins in all lanes.

d, e) Comparison of the expression level of ERGIC-53 (d) and secreted and expressed AAT-HiBiT (e). HEK293T cells transfected with AAT-HiBiT and empty vector, and ERGIC-53 KO 293T cells transfected with AAT-HiBiT and ERGIC-53, indicated mutant or empty vector were incubated in the serum-free Expi293 medium for 4h. Conditioned media and cell lysates were resolved by non-reducing (d) or reducing (e) SDS-PAGE and analyzed with immunoblotting with anti-ERGIC-53 antibody and LgBiT proteins for HiBiT-tagged proteins.

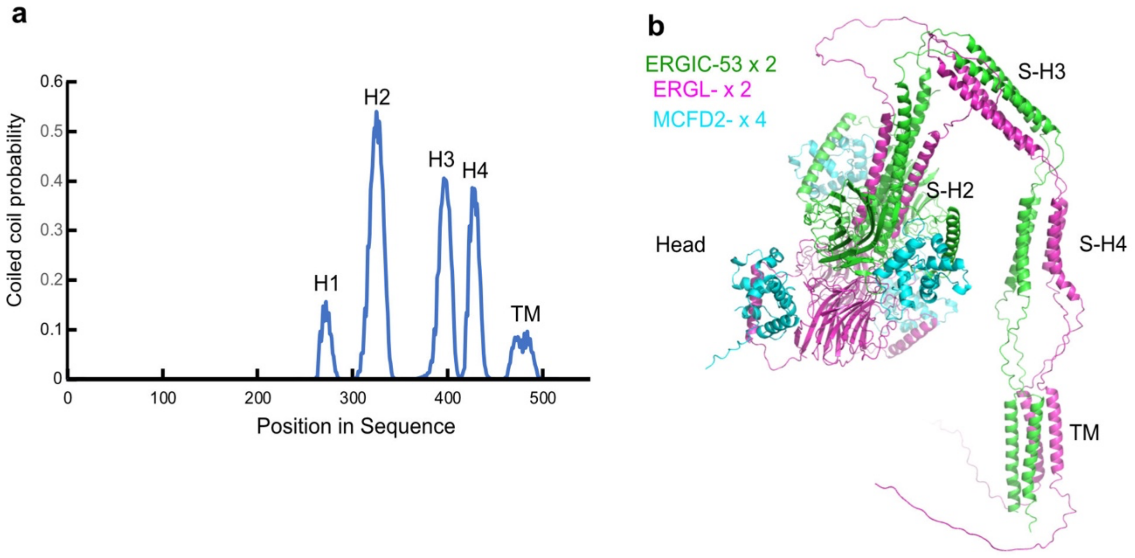

Supplementary Fig. 14 Prediction of ERGL structures

a) Prediction of the coiled-coil region of ERGL using DeepCoil2. The three stalk helices of ERGL (S-H2, S-H3 and S-H4) show a relatively high probability of coiled-coil formation, as the case for ERGIC-53.

b) A predicted model of a possible heterotetramer composed of two ERGIC-53 and two ERGL protomers complexed with four MCFD2, generated by Alphafold2 multimer.

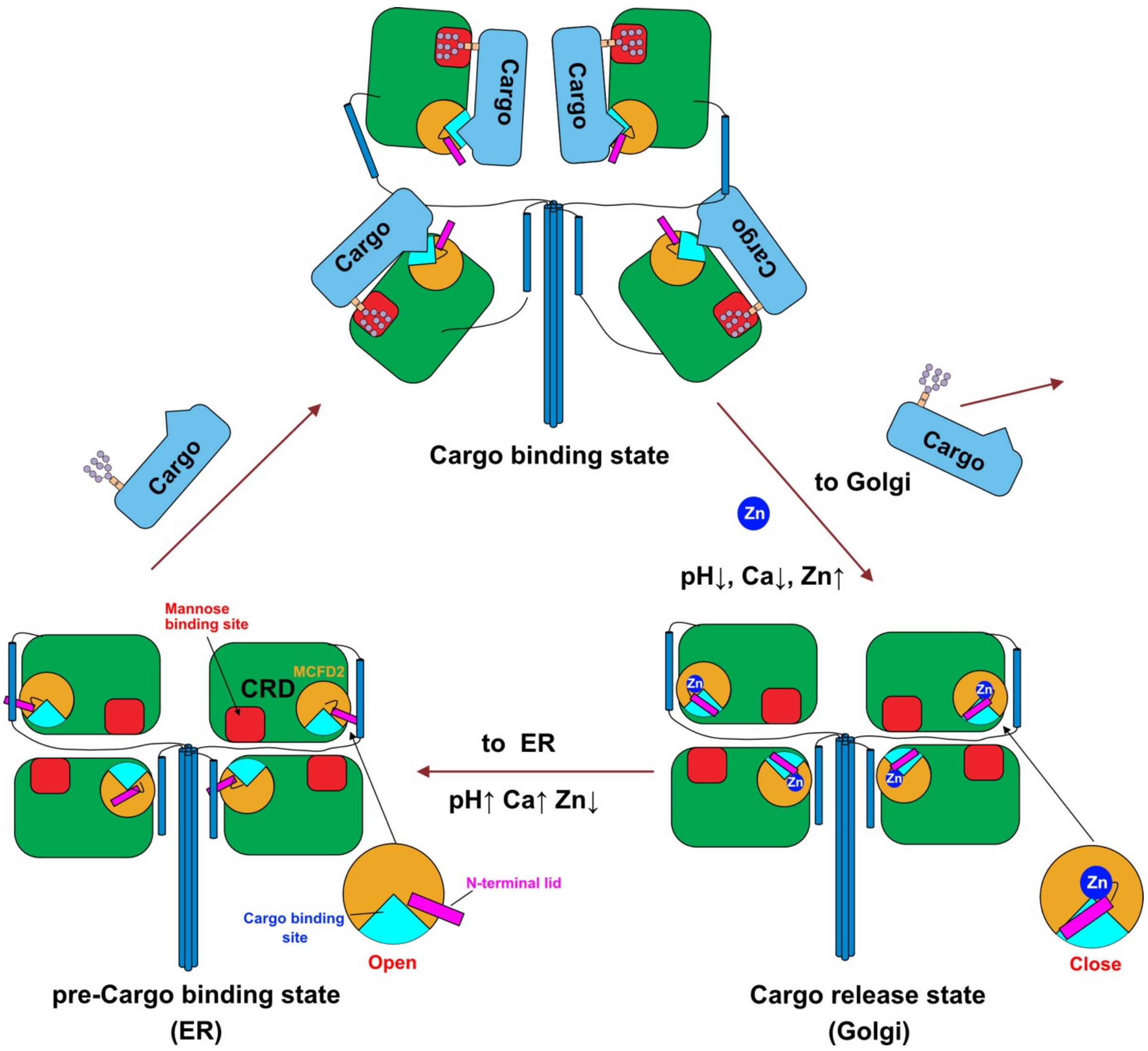

Supplementary Fig.15 A working model of Zn^{2+}, Ca^{2+} and pH-dependent regulation of cargo capture and release by the ERGIC-53 and MCFD2 complex (see text).

Supplementary Table 1 SAXS data acquisition, sample details, data analysis

**A. Sample details**		
	ERGIC-53	ERGIC-53-MCFD2 complex
Organism	Homo sapience	
Source	*HEK293T/ E. coli* expressed	
Uniprot ID	P49257, Q8NI22	
Extinction coefficient (A_{280nm}, Abs $_{0.1\%}$ (w/v))	0.851	0.766
MW from chemical composition (Da)	55350.14	69287.14
Initial concentration,for Injection (mg ml^{-1})	1.6	1.7
Injection Volume (μL), flow rate (ml/min)	50, 0.03	50, 0.03
concentration, (mg ml^{-1})	0.23-0.462	0.261-0.501
Concentration method	UV-Vis. spectroscopy	
Solvent composition	20 mM Tris-HCl, 150 mM NaCl, pH 7.5 10mM CaCl2, 0.02%GDN,	
**B. SAS data collection parameters**		
Source, instrument	Photon Factory BL-15A2	
Wavelength (Å)	1.0	
Camera Length (mm)	2650	
Beam geometry (μm)	V150 × H540 KB mirror + 2 slits + 1 pinhole	V150 × H540 KB mirror + 2 slits + 1 pinhole
q-measurement range (Å^{-1})	0.0077-0.319	0.0077-0.319
Absolute scaling method	Comparison with scattering from pure H_2O	
Basis for normalization to constant counts	Normalized to incident intensity by ion chamber	
Method for monitoring radiation damage	data frame-by-frame comparison	
Expo. time, No. of image	3 sec, 1600	3 sec, 1600
path length	1 mm	
Sample temperature (K)	RT	
**C. Software employed for SAS data reduction, analysis and interpretation**		
SAS data processing	*SAngler, MOLASS*	
Basic analyses: (Guinier, P(r))	*AUTORG PRIMUSqt*	
**D. Structural parameters**		
	ERGIC-53	ERGIC-53-MCFD2 complex
Guinier Analysis		
I(0) (cm^{-1})/Conc.	0.665 +/- 0.006	0.702 +/- 0.006
R_g (Å)	101.5 +/- 2.5	102.8+/- 4.1
q-range (Å^{-1})	0.007748-0.012642	0.008972-0.0118266
($q \times R_g$)	0.79-1.28	0.92-1.22
P(*r*) analysis		
I(0) (cm^{-1})/Conc.	0.682 +/- 0.01	0.727 +/- 0.02
R_g (Å)	108.7 +/- 2.1	110.8 +/- 2.2
D_{max} (Å)	347	350
q-range (Å^{-1})	0.0077-0.0787	0.0090-0.0775

Supplementary Table 2
Cryo-EM data collection, refinement and validation statistics

	Head region (form A) (EMDB-36467) (PDB 8JP4)	Head region (form B) (EMDB-36467) (PDB (8JP5)	Head region (substate A) (EMDB-36468) (PDB 8JP6)	Head region (substate B) (EMDB-36469) (PDB 8JP7)
**Data collection and processing**				
Magnification	105,000	105,000	105,000	105,000
Voltage (kV)	300	300	300	300
Electron exposure (e–/Å^2)	47.4	47.4	47.4	47.4
Defocus range (μm)	-0.8~ -1.6	-0.8~ -1.6	-0.8~ -1.6	-0.8~ -1.6
Pixel size (Å)	0.83	0.83	0.83	0.83
Symmetry imposed	C2	C2	C1	C1
Initial particle images (no.)	2,132,031	2,132,031	2,132,031	2,132,031
Final particle images (no.)	89361	81444	9734	9783
Map resolution (Å)	2.53	2.59	3.29	3.51
FSC threshold (0.143)				
Map resolution range (Å)	2.4~4.8	2.4~4.8		
**Refinement**				
Initial model used (PDB code)	3WNX			
Model resolution (Å)	3.0	3.2	3.3	3.5
FSC threshold (0.5)				
Model resolution range (Å)				
Map sharpening *B* factor (Å^2)	-68.9	-73	-65	-55
Model composition				
Non-hydrogen atoms	13500	13490	13500	13476
Protein residues	1678	1676	1678	1674
Ligands	20	20	20	20
B factors (Å^2)				
Protein	185.8	185.8	180.4	254.5
Ligand	132.31	132.8	144	202.9
R.m.s. deviations				
Bond lengths (Å)	0.002	0.003	0.003	0.005
Bond angles (°)	0.582	0.577	0.593	0.636
Validation				
MolProbity score	1.53	1.77	1.48	1.61
Clashscore	5.4	5.03	4.45	5.64
Poor rotamers (%)	1.8	2.35	0.14	0.62
Ramachandran plot				
Favored (%)	97.8	96.6	96.2	95.7
Allowed (%)	2.2	3.4	3.8	4.3
Disallowed (%)	0.0	0.0	0.0	0.0

	Head region (substate C) (EMDB-36471) (PDB 8JP8)	Head region (Substate D) (EMDB-36472) (PDB (8JP9)	Full length (EMDB-36479) (PDB 8JPG)	ΔH34 mutant (EMDB-36482)
**Data collection and processing**				
Magnification	105,000	105,000	105,000	60,000
Voltage (kV)	300	300	300	300
Electron exposure (e–/Å^2)	47.4	47.4	47.4	50
Defocus range (μm)	-0.8~ -1.6	-0.8~ -1.6	-0.8~ -1.6	-0.8 - -1.6
Pixel size (Å)	0.83	0.83	0.83	0.788
Symmetry imposed	C1	C1	C1	C1
Initial particle images (no.)	2,132,031	2,132,031	168,458	1,279,058
Final particle images (no.)	10,713	13,895	29762	138,608
Map resolution (Å)	3.39	3.37	6.72	3.78
FSC threshold (0.143)				
Map resolution range (Å)				
**Refinement**				
Initial model used (PDB code)				
Model resolution (Å)	3.4	3.4	8.9	
FSC threshold (0.5)				
Model resolution range (Å)	-54	-56		
Map sharpening *B* factor (Å^2)				
Model composition				
Non-hydrogen atoms	13500	13500	18068	
Protein residues	1678	1678	2252	
Ligands	20	20	20	
B factors (Å^2)				
Protein	214.1	218.8	1179.6	
Ligand	221.1	185.6	1124.6	
R.m.s. deviations				
Bond lengths (Å)	0.002	0.03	0.004	
Bond angles (°)	0.516	0.507	0.781	
Validation				
MolProbity score	1.2	1.19	1.87	
Clashscore	2.78	2.44	11.0	
Poor rotamers (%)	0.07	0.14	0.1	
Ramachandran plot				
Favored (%)	97.3	97.1	95.6	
Allowed (%)	2.7	2.9	4.4	
Disallowed (%)	0.0	0.0	0.0	

Supplementary Table 3 Primers used in this work

**Name**	**Sequence (5'-3')**
EGfw	AGCATCGATgccaccATGGCGGGATCCAGGCAAAGGGGTCTCCGG
EGrv	tggcatggcaacgcc AAAGAATTTTTTGGCAGCTGCTTCTTGCTGAG
EG53PA-pig1fw	GTTTGGtctagagctagcATCGATgccaccATGGCGG
EG53PA-RV1	gggagaggggcGCGGCCGCtcattacaccacatcatc
EG53_delH34_Fw2	AATCTCTTCAAATGAAAAGCCGAAATGC
EG53_delH34_rv2	TTCATTTGAAGAGATTTCCTCTGTTAAGG
EG53_delL1_Fw1	CCGACCTC AGTGTAGGAGATCGAGAG
EG53_delL1_rv1	CTACACTGAGGTCGGGGTGGCCCTTCTG
EG53W67Sfv	CCCTTCTCCGCCCACGCGGGGAATGCT
EG53W67Srv	GTGGGCGGAGAAGGGCACGGTCCCGTC
EG53-FLAGfw	AGCATCGATgccaccATGGCGGGATCCAGGCAAAGGGGTC
EG53-FLAGrv	gtcatccttgtaatcAAAGAATTTTTTGGCAGCTGCTTCTTG
PA_EG53_FW	tgccaggtgccgaagatgatgtggtgGACGGCGTGGGAGGAGACCCCGCGGTC
PA_EG53_RV	AGCGGCCGCtcattaAAAGAATTTTTTGGCAGCTGCTTCTTG
pcDNA-NPA-Fw	taatgaGCGGCCGCTCTAGAGGGCCCGTT
pcDNA-NPA-rv	tcttcggcacctggcatggcaacgccGCCCCGGACGAAGCGACCGAGTGACAG
EG53_pebFLAGFW1	gacggatccgccaccATGGCGGGATCCAGGCAAAGGGGTC
EG53_pebFLAGrv1	GTCATCCTTGTAATCAAAGAATTTTTTGGCAGCTGCTTCTTGC
peBFLAG_rv1	ggtggcggatccgtcgacctcgag
peBFLAG_fw1	GATTACAAGGATGACGACGATAAGTAATG
EG53_gRNAfw	CTTGGGCGGTTTGTACGGGGCggcgttgccatgccaggt
EG53_gRNArg	TACAAACCGCCCAAGTGACAGCAGCAAGGCGCAGAACAGCG
phlsec_fw1	gctagcgatatccctacgacgtgcccg
phlsec_rv1	agggatatcgctagcttacttatcgtcgtcatccttgtaatc
phLsecF5_fw2	gttgcgtagctgaaaccggtGCACAGCTAAGGCAGTTCTACGTGGCTG
phlsecF5_hibit_rv3	ATCACAGCCAAAGAGTTCCAGGCGAAG
