# Supplementary material for: Structure of full-length ERGIC-53 in complex with MCFD2 for cargo transport
Source: Nat Commun. 2024 Mar 16;15:2404. doi: 10.1038/s41467-024-46747-1 (PMC10944485; doi:10.1038/s41467-024-46747-1)
Supplement: Supplementary file 3 — Description of Additional Supplementary Files [file 41467_2024_46747_MOESM3_ESM.pdf]

Description of Additional Supplementary Files

File name: Supplementary Movie 1
Description: 3D variability analysis of full-length ERGIC-53 in complex with MCFD2 reveals the two different conformational motions of the stalk region.

File name: Supplementary Movie 2
Description: 3D variability analysis of the head region of full-length ERGIC-53 reveals the two different conformational motions of the CRDs.

File name: Supplementary Movie 3
Description: 3D variability analysis of ERGIC-53 ΔH34 in complex with MCFD2 reveals the three different conformational motions of this mutant.
