# Supplementary material for: Structure of full-length ERGIC-53 in complex with MCFD2 for cargo transport
Source: Nat Commun. 2024 Mar 16;15:2404. doi: 10.1038/s41467-024-46747-1 (PMC10944485; doi:10.1038/s41467-024-46747-1)
Supplement: Supplementary file 7 — Reporting Summary [file 41467_2024_46747_MOESM7_ESM.pdf]

nature portfolio

Corresponding author(s): Satoshi Watanabe, Kenji Inaba

Last updated by author(s): Feb 18, 2024

Reporting Summary

Nature Portfolio wishes to improve the reproducibility of the work that we publish. This form provides structure for consistency and transparency in reporting. For further information on Nature Portfolio policies, see our Editorial Policies and the Editorial Policy Checklist.

Statistics

For all statistical analyses, confirm that the following items are present in the figure legend, table legend, main text, or Methods section.

n/a	Confirmed	
☐	☒	The exact sample size (*n*) for each experimental group/condition, given as a discrete number and unit of measurement
☐	☒	A statement on whether measurements were taken from distinct samples or whether the same sample was measured repeatedly
☐	☒	The statistical test(s) used AND whether they are one- or two-sided<br>*Only common tests should be described solely by name; describe more complex techniques in the Methods section.*
☒	☐	A description of all covariates tested
☒	☐	A description of any assumptions or corrections, such as tests of normality and adjustment for multiple comparisons
☐	☒	A full description of the statistical parameters including central tendency (e.g. means) or other basic estimates (e.g. regression coefficient) AND variation (e.g. standard deviation) or associated estimates of uncertainty (e.g. confidence intervals)
☐	☒	For null hypothesis testing, the test statistic (e.g. *F*, *t*, *r*) with confidence intervals, effect sizes, degrees of freedom and *P* value noted<br>*Give P values as exact values whenever suitable.*
☒	☐	For Bayesian analysis, information on the choice of priors and Markov chain Monte Carlo settings
☒	☐	For hierarchical and complex designs, identification of the appropriate level for tests and full reporting of outcomes
☒	☐	Estimates of effect sizes (e.g. Cohen's *d*, Pearson's *r*), indicating how they were calculated

Our web collection on statistics for biologists contains articles on many of the points above.

Software and code

Policy information about availability of computer code

Data collection	serialEM 3.7, 3.8
Data analysis	RELION 3.1, 4.0, cryoSPARC v3.3, CTFFIND4, phenix 1.20, Coot 0.9.8, Pymol v2.5, Servalcat, Chimera X 1.5, Topaz 2.3, Jalview 1.8.3, Deepcoil2, SamCC, Graphpad prism 9.3,Colabfold 1.5

For manuscripts utilizing custom algorithms or software that are central to the research but not yet described in published literature, software must be made available to editors and reviewers. We strongly encourage code deposition in a community repository (e.g. GitHub). See the Nature Portfolio guidelines for submitting code & software for further information.

Data

Policy information about availability of data

All manuscripts must include a data availability statement. This statement should provide the following information, where applicable:

- Accession codes, unique identifiers, or web links for publicly available datasets
- A description of any restrictions on data availability
- For clinical datasets or third party data, please ensure that the statement adheres to our policy

The cryo-EM density maps have been deposited in the Electron Microscopy Data Bank (EMDB) under accession codes EMD-36467 (form A), EMD-36468 (form B), EMD- 36469 (substate A), EMD-36470 (substate B), EMD- 36471 (substate C), EMD-36472 (substate D), EMD- 36479 (full length) and EMD- 36482 (the ΔH34 mutant). The atomic coordinates have been deposited in the Protein Data Bank under accessions 8JP4 (form A), 8JP5 (form B), 8JP6 (substate A), 8JP7 (substate B),

8JP8 (substate C), 8JP9 (substate D), and 8JPG (full length). Raw movies have been deposited in EMPIAR-11645 (the ΔH34 mutant) and EMPIAR-11646 (full-length ERGIC-53). Other PDB codes used in this work. 3WNX (the crystal structures of the CRD and MCFD2 complex); 4YGE(Zn-free MCFD2 structure)

Research involving human participants, their data, or biological material

Policy information about studies with human participants or human data. See also policy information about sex, gender (identity/presentation), and sexual orientation and race, ethnicity and racism.

Reporting on sex and gender	Not Applicable
Reporting on race, ethnicity, or other socially relevant groupings	Not Applicable
Population characteristics	Not Applicable
Recruitment	Not Applicable
Ethics oversight	Not Applicable

Note that full information on the approval of the study protocol must also be provided in the manuscript.

Field-specific reporting

Please select the one below that is the best fit for your research. If you are not sure, read the appropriate sections before making your selection.

☒ Life sciences ☐ Behavioural & social sciences ☐ Ecological, evolutionary & environmental sciences

For a reference copy of the document with all sections, see nature.com/documents/nr-reporting-summary-flat.pdf

Life sciences study design

All studies must disclose on these points even when the disclosure is negative.

Sample size	No statistical method was used to determine the sample size. For the cryo-EM analyses, sample sizes were determined based on the sufficient number of particles to obtain structures at high resolution. For the secretion analysis, the sample size (n=3 or 4) was chosen to ensure the statistical reliability.
Data exclusions	No data was excluded.
Replication	For the cryo-EM analyses, structure determination was performed once, because the determined structures represent averaged structures of 10-90 k particles from a single data set, at sufficient resolution. For the FV and AAT secretion assays, the experiments were performed independently at least three times. All attempts at replication were successful.
Randomization	For the cryo-EM analyses, the particles were randomly classified into two groups for reconstruction of half maps to estimate the resolution of the maps. The secretion assay did not include experimental group allocation, therefore randomization was not performed
Blinding	Not applicable because this work did not contain experiments with group allocation.

Reporting for specific materials, systems and methods

We require information from authors about some types of materials, experimental systems and methods used in many studies. Here, indicate whether each material, system or method listed is relevant to your study. If you are not sure if a list item applies to your research, read the appropriate section before selecting a response.

Materials & experimental systems

n/a	Involved in the study	
☐	☒	Antibodies
☐	☒	Eukaryotic cell lines
☒	☐	Palaeontology and archaeology
☒	☐	Animals and other organisms
☒	☐	Clinical data
☒	☐	Dual use research of concern
☒	☐	Plants

Methods

n/a	Involved in the study	
☒	☐	ChIP-seq
☒	☐	Flow cytometry
☒	☐	MRI-based neuroimaging

Antibodies

Antibodies used	Anti-LMAN1 antibody (abcam, ab125006), anti-Factor V(Abcam, ab108614)
Validation	https://www.abcam.co.jp/products/primary-antibodies/lman1-antibody-epr6979-ab125006.pdf, https://www.abcam.co.jp/products/primary-antibodies/factor-v-antibody-epr5191-ab108614.pdf

Eukaryotic cell lines

Policy information about cell lines and Sex and Gender in Research

Cell line source(s)	HEK293T (ATCC CRL-3216), HEK293T LMAN1 KO cells (Abcam, ab266248), HEK293 WT cells (Abcam, ab255449).
Authentication	None of the cell lines used in this work have been authenticated.
Mycoplasma contamination	Not tested.
Commonly misidentified lines (See ICLAC register)	No commonly misidentified lines were used in this study

Plants

Seed stocks	N.A
Novel plant genotypes	N.A
Authentication	N.A
